# Accounting for Uncertainty in the Null Benchmark in Two-Stage Phase II Trials

**DOI:** 10.64898/2026.05.14.26353210

**Published:** 2026-05-18

**Authors:** Rebecca Irlmeier, Zhuoli Jin, Fei Ye

## Abstract

**Background:**

Simon two-stage designs for binary endpoints and their time-to-event analogues, including the Kwak and Jung method, rely on a fixed null benchmark. Their Type I error control is valid only when that benchmark is correctly specified. In practice, historical benchmarks are often inconsistent due to small samples, population heterogeneity, changing eligibility criteria, and evolving standards of care. Even modest misspecifications can substantially inflate the Type I error rate, leading to costly advancement of ineffective treatments.

**Methods:**

We propose the Interval-Null Robust (INR) two-stage design framework that accounts for uncertainty in the historical null benchmark. We define the null hypothesis as a plausible range of clinically uninteresting values: *p* ∈ [ *p*_ 0 *L*_ , *p*_ 0 *U*_ ] for binary endpoints and *λ* ∈ [ *λ*_ 0 *L*_ , *λ*_ 0 *U*_ ] (or equivalent survival probabilities) for time-to-event endpoints. Type I error is controlled uniformly over the full null interval: . Under the monotonicity of the Go probability, the supremum occurs at the least favorable null configuration – *p*_ 0 *U*_ and *λ*_ 0 *L*_ – but the design is not reduced to a point-null formulation. The interval defines the uncertainty set for error control and is used in selecting among feasible designs through robust criteria such as worst-case regret or minimal average expected sample size.

**Results:**

Across representative planning scenarios for both endpoint types, classic designs calibrated to a single benchmark exhibit substantial Type I error inflation when the true null parameter exceeds the assumed planning value. INR designs maintain the nominal Type I error rate across the full null interval, directly addressing this vulnerability to benchmark misspecification. The robustness-efficiency trade-off can be managed through design constraints and robust optimization criteria while preserving uniform Type I error control.

**Conclusions:**

INR two-stage designs offer a transparent framework for addressing historical control uncertainty in single-arm Phase II trials. By replacing reliance on a fixed benchmark assumption with a more realistic interval of clinically plausible null values, INR design reduces the risk of false-positive Go-decisions caused by benchmark misspecification. INR applies to both binary and time-to-event endpoints and is implemented in the open-source **INRDesign** R package and accompanying interactive Shiny app.

## Full Text Availability

The license terms selected by the author(s) for this preprint version do not permit archiving in PMC. The full text is available from the preprint server.

